# Immunogenic cell death after combined treatment with radiation and ATR inhibitors is dually regulated by apoptotic caspases

**DOI:** 10.3389/fimmu.2023.1138920

**Published:** 2023-06-06

**Authors:** Adrian Eek Mariampillai, Sissel Hauge, Karoline Kongsrud, Randi G. Syljuåsen

**Affiliations:** ^1^ Department of Radiation Biology, Institute for Cancer Research, Norwegian Radium Hospital, Oslo University Hospital, Oslo, Norway; ^2^ Institute of Clinical Medicine, Faculty of Medicine, University of Oslo, Oslo, Norway

**Keywords:** immunogenic cell death (ICD), radiation therapy (radiotherapy), ATR, caspase, CALR (calreticulin), ATP - adenosine triphosphate, HMGB1 (high mobility group box 1)

## Abstract

**Introduction:**

Inhibitors of the ATR kinase act as radiosensitizers through abrogating the G2 checkpoint and reducing DNA repair. Recent studies suggest that ATR inhibitors can also increase radiation-induced antitumor immunity, but the underlying immunomodulating mechanisms remain poorly understood. Moreover, it is poorly known how such immune effects relate to different death pathways such as caspase-dependent apoptosis. Here we address whether ATR inhibition in combination with irradiation may increase the presentation of hallmark factors of immunogenic cell death (ICD), and to what extent caspase activation regulates this response.

**Methods:**

Human lung cancer and osteosarcoma cell lines (SW900, H1975, H460, U2OS) were treated with X-rays and ATR inhibitors (VE822; AZD6738) in the absence and presence of a pan-caspase inhibitor. The ICD hallmarks HMGB1 release, ATP secretion and calreticulin surface-presentation were assessed by immunoblotting of growth medium, the *CellTiter-Glo* assay and an optimized live-cell flow cytometry assay, respectively. To obtain accurate measurement of small differences in the calreticulin signal by flow cytometry, we included normalization to a barcoded control sample.

**Results:**

Extracellular release of HMGB1 was increased in all the cell lines at 72 hours after the combined treatment with radiation and ATR inhibitors, relative to mock treatment or cells treated with radiation alone. The HMGB1 release correlated largely – but not strictly – with loss of plasma membrane integrity, and was suppressed by addition of the caspase inhibitor. However, one cell line showed HMGB1 release despite caspase inhibition, and in this cell line caspase inhibition induced pMLKL, a marker for necroptosis. ATP secretion occurred already at 48 hours after the co-treatment and did clearly not correlate with loss of plasma membrane integrity. Addition of pan-caspase inhibition further increased the ATP secretion. Surface-presentation of calreticulin was increased at 24-72 hours after irradiation, but not further increased by either ATR or caspase inhibition.

**Conclusion:**

These results show that ATR inhibition can increase the presentation of two out of three ICD hallmark factors from irradiated human cancer cells. Moreover, caspase activation distinctly affects each of the hallmark factors, and therefore likely plays a dual role in tumor immunogenicity by promoting both immunostimulatory and -suppressive effects.

## 1 Introduction

Radiotherapy is a cornerstone of cancer treatment, but is often not sufficient for tumor ablation on its own. Hence, radiotherapy is typically combined with other treatment modalities. The serine/threonine protein kinase ATR, which regulates cell cycle checkpoints and DNA repair, is a promising target for such combination treatment. Cancer cells are often found to have a dysfunctional G1 checkpoint, rendering them more reliant on the G2 checkpoint (1, 2). Upon radiation-induced DNA damage, activated ATR is required for the S and G2 checkpoints and homologous recombination repair (3, 4). Inhibition of ATR activity will thus cause the cells to progress through mitosis with unrepaired DNA, resulting in more cell death *via* mitotic catastrophe (5). ATR inhibitors (ATRi) are thus acting as radiosensitizers (6, 7). Combined treatment with radiation and ATR inhibitors is currently tested in clinical trials (8, 9).

In addition to DNA damage and cell death, radiotherapy causes both immunogenic and immunosuppressive effects in the cancer microenvironment [reviewed in (10, 11)]. A major goal is to enhance and exploit the immunostimulatory properties of radiotherapy, in order to prime antitumor immunity and optimize combination with *e.g.* immune checkpoint blockade. However, the interaction between radiotherapy and the immune system is complex, and more knowledge is needed in order to fully understand its possibilities and limitations. Immunostimulatory effects of radiotherapy may *e.g.* be induced when irradiated cancer cells undergo immunogenic cell death (ICD) (12) [reviewed in (13, 14)]. ICD is defined as cell death with the potential to induce immune responses through presentation of damage-associated molecular patterns (DAMPs) (15, 16). The presentation of three such DAMPs have been established as major hallmarks for ICD, namely release of the non-histone nuclear protein high mobility group box-1 (HMGB1), secretion of adenosine 5’-triphosphate (ATP) and surface-presentation of the endoplasmic reticulum protein calreticulin (ecto-CALR) (17). When these DAMPs are presented on or from dying cancer cells, they act as adjuvants (or ‘danger signals’) (18), enabling dendritic cells of the immune system to recognize tumor-associated antigens (TAAs) as dangerous, and thus induce T cell responses towards the tumor cells (16).

Interestingly, recent preclinical studies suggest that ATR inhibition can increase the immunostimulatory effects of radiotherapy. This has been demonstrated in multiple murine models *in vivo*, where ATR inhibition combined with irradiation caused activation of CD8^+^ T cells, dendritic cells and natural killer cells, induction of immunological memory and less regulatory T cell-mediated immunosuppression (11, 19–21). Nevertheless, the underlying molecular mechanisms are incompletely understood. ATR inhibition may cause downregulation of programmed cell death 1 ligand 1 (PD-L1) and leukocyte surface antigen 47 (CD47), thereby giving a partial suppression of the PD-1/PD-L1 and SIRPα/CD47 immune checkpoints (22). ATR inhibition can also promote efferocytosis, where apoptotic tumor cells are engulfed by phagocytes such as dendritic cells (23). Moreover, ATR inhibition can increase type I interferon (IFN) signaling *via* induction of cytosolic DNA or RNA in irradiated tumor cells (24–26). ATR thus appears to regulate multiple immunomodulating mechanisms after irradiation. However, to our knowledge, it is not known whether ATR inhibition also affects radiation-induced expression of the abovementioned hallmark factors of ICD.

ICD may be linked to specific cell death mechanisms such as apoptosis, which is executed by activated caspases [reviewed in (27)]. Caspase activation has been shown to promote chemotherapy-induced ATP secretion and calreticulin surface-presentation (28, 29). On the other hand, caspases are generally associated with immunosuppression, as a part of the intended immunological silence of apoptosis [reviewed in (27)]. Apoptotic caspases may therefore also inhibit treatment-induced antitumor immune responses. They can for instance inhibit the mentioned type I IFN response through mediating cleavage of the cytosolic DNA sensor cGAS or other components of the cGAS–STING–IFN pathway [reviewed in (30, 31)]. In line with this, we recently showed that the IFN response to treatment with irradiation and ATR inhibition is counteracted by caspase activation (26). Apoptotic caspases also suppress the release of HMGB1 from mouse melanoma cells after irradiation (32), and may also indirectly inactivate HMGB1 (33). Furthermore, combining irradiation with caspase inhibition gives enhanced antitumor immune responses and tumor regression in murine tumor models *in vivo* (34–36). Caspase inhibition may thus be a potential strategy to enhance the immunostimulatory effects of radiotherapy.

In this study, we hypothesized that irradiation combined with ATR inhibition increases the extent of immunogenic cell death, as ATR inhibition abrogates the radiation-induced G2 checkpoint and disables DNA repair. We also hypothesized that caspase activation contributes to regulate ICD in this setting, in concordance with the previously reported chemotherapy-induced ICD mentioned above (28, 29). The results show that ATR inhibition can increase radiation-induced presentation of HMGB1 and ATP – two of the three ICD hallmark factors. This suggests that the combination treatment with irradiation and ATR inhibition may contribute to priming of antitumor immunity. Furthermore, we show that caspase inhibition has distinct effects on each of the ICD factors, and that caspase activation therefore may promote both immunostimulatory and -suppressive effects after the combined treatment.

## 2 Results

### 2.1 Combined treatment with radiation and ATR inhibitors triggers extracellular release of HMGB1 from human cancer cells

**Figure 1:**
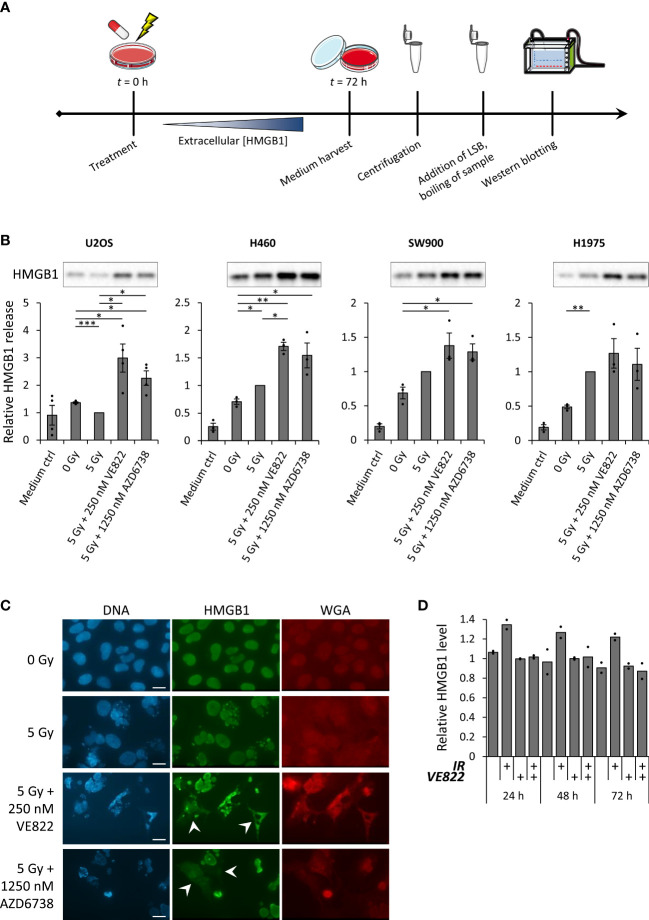
Combined treatment with radiation and ATR inhibitors translocate HMGB1 from the nucleus to the cytoplasm, and increase radiation-induced extracellular HMGB1 release. **(A)** Experimental set-up for measurement of HMGB1 release. Medium from treated cells was collected 72 hours post treatment to allow for accumulation of extracellular HMGB1. After harvest, the samples were centrifuged in order to exclude floating cells, before they were diluted in loading sample buffer (LSB) and analyzed by SDS-PAGE and western blotting. **(B)** Results from three or more experiments performed as in **(A)** for U2OS, H460, SW900 and H1975 cells. Bar charts show quantification of extracellular HMGB1. In each experiment the values are normalized to the values for the 5 Gy treatment. *p* values were calculated as described in the Materials and methods section (not included for medium controls). Immunoblots on top of the bar charts are from a representative experiment. **(C)** Micrographs of U2OS cells stained with antibody against HMGB1 (green), the nuclear stain Hoechst (blue) and cell membrane staining, fluorochrome-conjugated WGA (red) at 72 hours post treatment. White arrows indicate cells with cytoplasmic HMGB1 signal. Scale bar = 20 µm. **(D)** Relative levels of intracellular HMGB1 in viable U2OS cells at indicated time-point after treatment with ionizing radiation (5 Gy; IR) and/or the ATR inhibitor VE822 (250 nM), as measured by flow cytometry. Cells were stained with Pacific Blue (PB) before fixation to distinguish between viable (PB^-^) and non-viable (PB^+^) cells. (Viable cells were gated as in **Supplementary Figure S2B**). *n* = 2. *p ≤ 0.05, **p ≤ 0.01, ***p ≤ 0.001.

In order to evaluate whether ATR inhibitors can increase the expression of ICD hallmark factors after irradiation, we first measured HMGB1 release by immunoblotting of growth medium harvested at 72 hours after treatment (**Figures 1A, B**). The human osteosarcoma cell line U2OS and the human non-small-cell lung cancer (NSCLC) cell lines H460, SW900 and H1975 were included in this analysis. We have previously observed increased IFN signaling in U2OS and the NSCLC cell lines at 72 hours after treatment with 5 Gy X-rays and the two ATR inhibitors VE822 and AZD6738 at concentrations of 250 nM and 1250 nM, respectively (26). We hence used the same radiation dose, ATR inhibitor concentrations and time-point as in the previous study. All cell lines showed similar kinetics of G2 checkpoint abrogation (26). They also showed increased amount of non-viable cells at 72 hours after the co-treatment with radiation and ATR inhibitors (**Supplementary Figure S1A**). We found that the co-treatment increased the presence of HMGB1 in the medium of samples from all the cell lines, relative to either the mock treatment or radiation treatment alone (**Figure 1B**). In addition, 5 Gy irradiation alone increased extracellular HMGB1 in two of the cell lines (H460 and H1975) and gave a non-significant increase in another (SW900) (**Figure 1B**). As the serum of the growth medium will contain bovine HMGB1, we included a medium control sample to our analysis, to verify that the signals were higher than the background HMGB1 level (**Figure 1B**). Of note, HMGB1 release was also increased by the co-treatment if cells were cultured in serum-free medium with the serum substitute B-27 (**Supplementary Figure S1B**). Timecourse analysis of U2OS cells showed that the release of HMGB1 did not occur much earlier than 72 hours after treatment, as it was not detected at 24-48 hours (**Supplementary Figure S1C**). This correlated with an increased amount of non-viable cells at 72 hours (**Supplementary Figure S1D**). Furthermore, a lower concentration of the ATR inhibitor VE822 (50 nM) did not yield detectable HMGB1 release (**Supplementary Figure S1C**).

The release of HMGB1 is believed to occur in a two-step process. First, the HMGB1 translocates from its primary location in the nucleus to the cytoplasm (37). From here, the HMGB1 can be actively secreted (38–40) or passively released [reviewed in (41)] over the cell membrane to the extracellular space. As the immunoblotting of HMGB1 only measured the extracellular HMGB1, we used immunofluorescence microscopy to assess nuclear *versus* cytoplasmic HMGB1 localization following the combined treatment. Whereas HMGB1 was detected only in the nucleus of non-treated U2OS cells, HMGB1 was localized both to the nucleus and to the cytoplasm after treatment with ATR inhibition and irradiation, thus confirming transport of HMGB1 from the nucleus to the cytoplasm (**Figure 1C**). We next wanted to assess whether the subsequent extracellular release of HMGB1 only occurred from cells with disintegrated cell membranes. To test this, we measured the levels of remaining intracellular HMGB1 in viable *versus* non-viable U2OS cells after treatment. The cell samples were viability-stained with Pacific Blue (PB), before formalin fixation, staining with an anti-HMGB1 antibody and flow cytometry analysis (**Supplementary Figure S2A**). A barcoded mock sample was added to all samples for accurate quantification of HMGB1 levels. The overall levels of intracellular HMGB1 in viable cells (PB^-^) were not markedly reduced after any of the treatments (**Figure 1D**) and were generally higher than for non-viable cells (**Supplementary Figure S2B**; bottom histograms). We noted that irradiation alone caused a slight increase in intracellular HMGB1 (**Figure 1D**). This could likely be related to increased cell size, particularly at 24-48 hours after treatment when irradiated cells remain arrested at the G2 checkpoint. However, when examining the HMGB1 histograms from viable cells at 72 hours post treatment, a proportion of the viable cells (PB^-^) from samples treated with ATR inhibition and irradiation showed low HMGB1 levels comparable to the bulk population of non-viable cells (**Supplementary Figure S2B**). *Vice versa*, a proportion of the non-viable cells (PB^+^) showed high HMGB1 levels, comparable to the levels of the bulk population of viable cells (**Supplementary Figure S2B**, bottom). Taken together, these results suggest that HMGB1 release after treatment with ATR inhibition and irradiation occurs more frequently for non-viable than viable cells, but it is not strictly correlated with loss of membrane integrity.

### 2.2 Combined treatment with radiation and ATR inhibitors increases secretion of ATP

**Figure 2:**
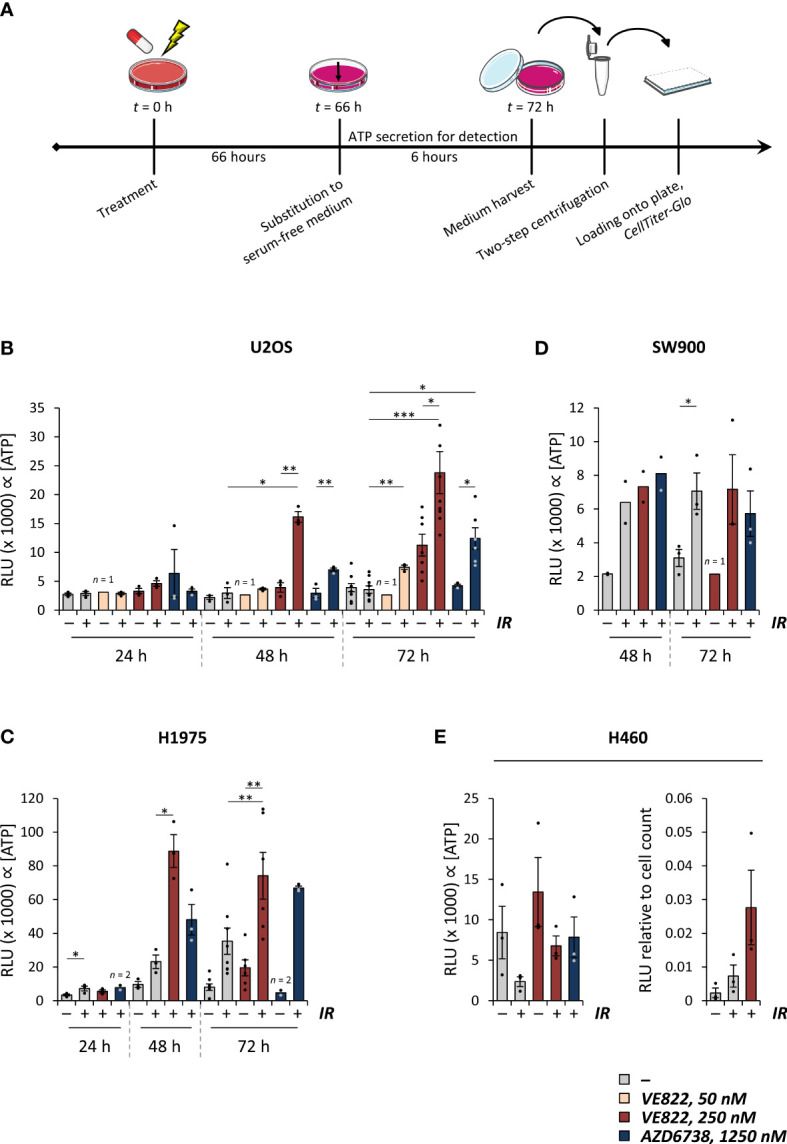
Combined treatment with radiation and ATR inhibitors increases ATP secretion. **(A)** Experimental set-up for measurement of ATP secretion, exemplified for the 72 hour time-point. Treated samples were incubated for 72 hours. Six hours prior to medium harvest, the medium was replaced with a reduced amount of serum-free medium. The media were collected from the samples, and centrifuged twice for cell exclusion, before the medium supernatants were analyzed by use of the *CellTiter-Glo* assay. Same set-up was employed for the other time-points, still with medium replacement for the last six hours. **(B-D)** Results from experiments performed as in **(A)** for U2OS **(B)**, H1975 **(C)** and SW900 cells **(D)** at indicated time-points after treatment with ionizing radiation (5 Gy; IR) and/or ATR inhibitors (VE822 or AZD6738 at indicated concentration). Bar charts show crude relative luminescence values (relative luminescence units; RLU), which is proportional to the extracellular concentration of ATP. *p* values were calculated for difference between co-treatments and irradiation alone or ATR inhibition alone. For statistical analysis between groups of different size, only data that paired up from the same experiments were included. **(E)** Results from experiments performed as in **(A)** in H460 cells, presented as for **(B-D)** (left). To correct for vast differences in cell count after treatment in this cell line, the relative luminescence values were normalized to cell counts at time of harvest (right). *p ≤ 0.05, **p ≤ 0.01, ***p ≤ 0.001.

To measure ATP secreted to the growth medium, we treated cell samples and incubated them for 24-72 hours. As serum may contain ATPases that can perturb the ATP measurements, we replaced the growth medium with fresh, serum-free medium six hours prior to medium harvest (**Figure 2A**). *CellTiter-Glo* measurements of the harvested medium samples revealed an increase in ATP secretion at 48-72 hours after irradiation alone in H1975, SW900 and H460, but not in U2OS cells (**Figures 2B–E**). The co-treatment led to increased secretion in U2OS, H1975 and H460 cells, as there was a higher secretion after the co-treatment compared to after treatment with radiation or ATR inhibitor alone in all experiments (**Figures 2B, C, E**). Timecourse analysis showed that the co-treatment increased ATP secretion at 48 and 72 hours, but not at 24 hours after treatment (**Figures 2B, C**). However, the ATP secretion was not increased after the co-treatment compared to after irradiation alone in SW900 cells (**Figure 2D**). Although ATP secretion was clearly most pronounced with the highest ATR inhibitor concentration of 250 nM VE822, a small increase in ATP secretion was also observed 72 hours after irradiation in combination with 50 nM VE822 (**Figure 2B**). Of note is that the results for H460 had to be normalized to the cell number of the dish at time of harvest, as the treatments severely impacted the cell growth relative to the rapidly dividing mock sample (**Figure 2E**). Altogether, these results show that ATP secretion is increased after irradiation alone in three of the cell lines, and increased relative to mock in all cell lines after the co-treatment. Interestingly, the ATP secretion is markedly increased already at 48 hours after the co-treatment.

### 2.3 An optimized ecto-CALR detection protocol reveals increased ecto-CALR after irradiation, but no further increase after combined treatment with radiation and ATR inhibitors

**Figure 3:**
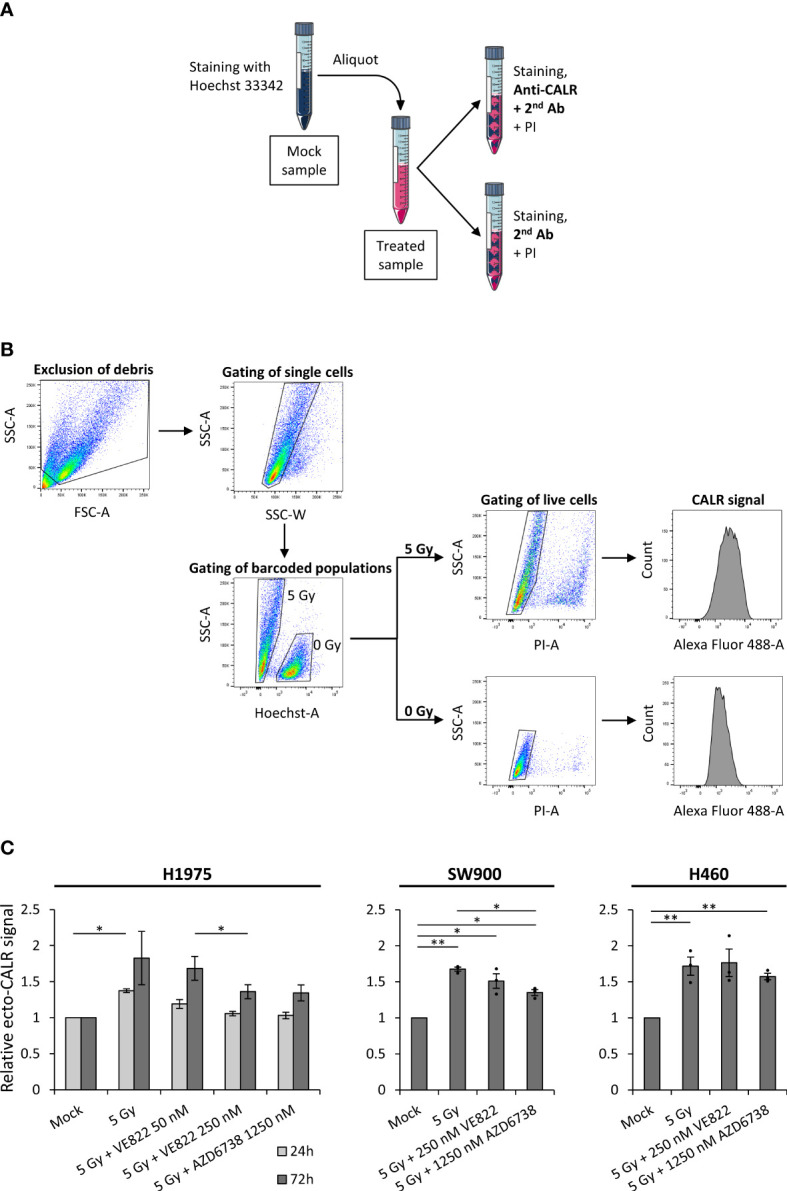
Surface-presentation of calreticulin is increased upon irradiation, but is not further increased by the addition of ATR inhibitors. **(A)** Procedure of Hoechst 33342-based barcoding, in which a live mock sample was stained with permeable Hoechst 33342, before it was divided equally to differently treated live-cell samples. Each of the barcoded samples were thereafter split into two aliquots; one for primary anti-calreticulin (anti-CALR) staining and one for secondary antibody control staining. The two aliquots were thereafter stained with propidium iodide (PI) for discrimination of live and dead cells. **(B)** The gating hierarchy employed for the flow cytometry analysis. Debris was excluded and cells were gated in a forward-scatter area (FSC-A) *versus* side-scatter area (SSC-A) plot. Cell singlets were gated in a side-scatter width (SSC-W) *versus* SSC-A plot. The barcoded populations were separated in a Hoechst-A *versus* SSC-A plot, in which the Hoechst 33342-stained mock population is shifted upwards the Hoechst-A axis. Live cells were gated in PI-A *versus* SSC-A plots, in which dead (PI^+^) cells are shifted upwards the PI-A axis. Finally, histograms of the ecto-CALR signals (Alexa Fluor 488) are obtained from the live cells in both of the barcoded populations, and the median value of ecto-CALR signal is obtained from each histogram. Similar gating hierarchy and analysis was done for the secondary antibody control samples. (Demonstrated in H1975 cells). **(C)** Results from experiments performed as in **(A, B)** for H1975, SW900 and H460 cells after treatment with ionizing radiation and ATR inhibitors. Bar charts show ecto-CALR signals normalized to the barcoded mock population of each sample. Note that results from both a 24 hours time-point (light grey) and 72 hours time-point (dark grey) are shown for H1975, whereas results from the 72 hours time-point are shown for SW900 and H460. *p ≤ 0.05, **p ≤ 0.01.

Cells undergoing ICD may translocate calreticulin from the endoplasmic reticulum to the cell surface. Nevertheless, the increase in ecto-CALR might be small, making accurate detection important. We therefore optimized a live-cell flow cytometry-based detection protocol, in which we included a barcoding strategy to eliminate any variation that might occur due to differences in antibody staining between samples. First, a mock sample, consisting of non-treated cells, was stained with cell permeable Hoechst 33342 and distributed in equal portions to the samples with treated, non-stained cells. Thereafter, the barcoded samples were stained with anti-CALR and secondary antibodies, as well as the non-permeable DNA-stain propidium iodide for live/dead cell differentiation (**Figure 3A**). In this way, the Hoechst-stained mock sample served as an internal standard enabling normalization of the ecto-CALR signals from all treated samples to the ecto-CALR signal of a common mock sample. The mock and treated cell populations were separated by the Hoechst 33342 signal during data analysis (**Figure 3B**). All of the barcoded samples were split into secondary antibody controls as well, and we performed similar analysis for these secondary antibody controls. Hence, we could subtract the background signals of the secondary antibody controls from the ecto-CALR signals (**Supplementary Figure S3**). Importantly, the background signals of the secondary antibody controls were shifted upon the various treatments, and it was therefore crucial to include secondary antibody controls for all samples in the experiment.

Using this method, we found that irradiation alone (5 Gy) increased ecto-CALR presentation by a factor of ~1.5 at 24 hours post treatment and ~1.8 at 72 hours post treatment relative to mock in H1975 (**Figure 3C**). No further increase was seen after the co-treatment with radiation and ATR inhibitors (**Figure 3C**). Rather, the co-treatment showed a trend towards reduction in radiation-induced ecto-CALR presentation in H1975. Similarly, we observed an increase in ecto-CALR at 72 hours post irradiation for SW900 and H460, but no further increase after the co-treatment (**Figure 3C**). U2OS cells were not included in this assay as the ecto-CALR signals were too low to be distinguished from the secondary antibody controls in this cell line (data not shown). We conclude that our optimized flow cytometry assay reveals a small increase in ecto-CALR after irradiation alone, but no further increase after co-treatment with irradiation and ATR inhibition.

### 2.4 Inhibition of apoptotic caspases differentially modulates the HMGB1 release, ATP secretion and ecto-CALR after combined treatment with radiation and ATR inhibition

**Figure 4:**
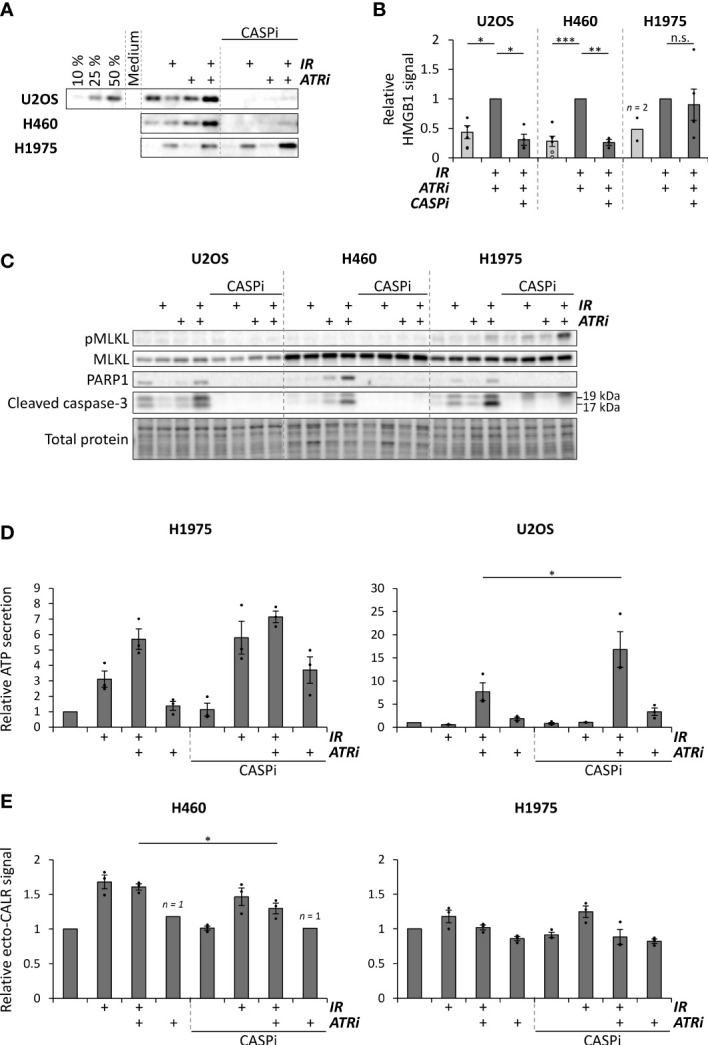
Caspase inhibition can suppress HMGB1 release, increases ATP secretion and does not alter ecto-CALR presentation after combined treatment with radiation and ATR inhibitors. **(A)** Representative immunoblots of extracellular HMGB1 in medium supernatants from U2OS, H460 and H1975 cells at 72 hours after treatments with 5 Gy radiation (IR) and/or 250 nM VE822 (ATRi) and 10 µM of the pan-caspase inhibitor Q-VD-OPh (CASPi). Gradient in the U2OS blot represents different volumes loaded from the co-treated sample. **(B)** Quantification of extracellular HMGB1 in independent experiments performed as for the immunoblots in **(A)**, from samples treated with 5 Gy or 6 Gy (IR; black and grey dots, respectively), 250 nM VE822 (ATRi) and 10 µM Q-VD-OPh (CASPi), normalized to the sample treated with IR + ATRi. (Please note that 1, 3, 2 and 3 data-points for the mock of U2OS and H1975 and the triple-treatment for U2OS and H460, respectively, were non-detectable and hence excluded from the quantification. Averages for these treatments were therefore even lower in reality). n.s. = non-significant. **(C)** Immunoblots of cleaved caspase-3, cleaved PARP1, phosphorylated MLKL and total MLKL in cell lysates corresponding to the supernatants used for the HMGB1 immunoblots in **(A)**. **(D)** ATP secretion in H1975 and U2OS cells with and without caspase inhibitor Q-VD-OPh (CASPi, 10 µM), normalized to the mock sample. ATP secretion was measured at 72 hours post treatment as in **Figure 2A**. **(E)** Results from the ecto-CALR flow cytometry assay in H460 and H1975 cells treated for 72 hours with and without 10 µM Q-VD-OPh (CASPi). Values are normalized to the barcoded mock signal (0 Gy), as in **Figure 3**. *p ≤ 0.05, **p ≤ 0.01, ***p ≤ 0.001.

We have previously shown that activated caspases suppress IFN-β secretion after the co-treatment with irradiation and ATR inhibition (26). To assess whether caspase activation also affects ICD after irradiation and ATR inhibition, we used the inhibitor Q-VD-OPh, which inhibits several caspases including the apoptotic caspases 3, 7, 8 and 9. In contrast to the effects on IFN-β secretion, we found that the caspase inhibitor strongly suppressed the HMGB1 release in H460 and U2OS cells, suggesting that the HMGB1 release is coupled to caspase activity and apoptosis (**Figure 4A, B**). In line with a specific role of apoptotic caspases in this process, the HMGB1 release was not much affected by two inhibitors of caspase-1 that did not suppress caspase-3 cleavage (**Supplementary Figure S4A**). Notably, Q-VD-OPh did not appear to inhibit HMGB1 release in H1975 cells, despite inhibition of caspase-3 cleavage (**Figure 4A-C**; **Supplementary Figure S4B**). However, in this cell line caspase inhibition caused increased phosphorylation of the pseudokinase mixed lineage kinase domain-like protein (pMLKL), a marker for necroptosis (**Figure 4C**). Necroptosis has previously been linked to HMGB1 release after caspase inhibition (32). The HMGB1 release after caspase inhibition in H1975 is thus likely caused by redirection of cell death towards necroptosis. On the other hand, the ATP secretion measured by the *CellTiter-Glo* assay was actually increased upon addition of Q-VD-OPh in U2OS and slightly increased in H1975 (**Figure 4D**), the two cell lines with highest increase in **Figure 2**. Caspase inhibition thus gave opposite effects on HMGB1 release and ATP secretion in U2OS cells. Caspase inhibition showed no major effects on the ecto-CALR signal in either H1975 or H460 cells (**Figure 4E**). (As mentioned above, U2OS was not included in the ecto-CALR measurements as the signal was too low). During flow cytometry analysis, we also quantified the percentage of live cells based on the exclusion of propidium iodide positive cells. As expected, the caspase inhibitor partly rescued the decrease in cell viability seen upon the co-treatment with irradiation and ATR inhibition (**Supplementary Figure S4C**). Altogether, these results show that treatment-induced caspase activation gives distinct effects on each of the ICD hallmark factors, as well as on IFN-β signaling, thus likely promoting both immunostimulatory and -suppressive effects.

## 3 Discussion

In this study, we have assessed presentation of three hallmark factors for immunogenic cell death – namely release of HMGB1, secretion of ATP and surface-presentation of CALR – in human cancer cell lines after treatment with radiation and ATR inhibitors. To our knowledge, this is the first study reporting whether ATR inhibition can increase the radiation-induced expression of these ICD factors. We found that the combined treatment with radiation and ATR inhibitors can increase the release of HMGB1 and secretion of ATP, but not surface-presentation of CALR, in several human cancer cell lines. Previous studies have shown that ATR inhibition affects multiple immunomodulating mechanisms after irradiation, including type I IFN responses, efferocytosis and immune checkpoints (22–26). Our new results suggest that increased HMGB1 release and ATP secretion may be added to this list of immunomodulating mechanisms that promote antitumor immunity after the combined treatment.

Moreover, our results show that activated caspases can modulate the ICD response after treatment with irradiation and ATR inhibition: Caspase inhibition can abolish the extracellular release of HMGB1, as shown for two cell lines, but increases the ATP secretion and does not alter the CALR surface presentation. We have previously reported that caspase inhibition strongly increases a type I IFN response after the combined treatment with radiation and ATR inhibitors (26). Caspase inhibition thus leads to distinct effects on each of these factors: It clearly exerts immunostimulatory effects on IFN signaling and also appears to promote immunostimulatory ICD through increased ATP secretion. Nevertheless, it contributes neither stimulatory nor suppressive through ecto-CALR presentation, and can exert immunosuppression through vastly reducing the HMGB1 release. An interesting issue for future studies is to determine which of these factors are most important for antitumor immunity, in order to evaluate the physiological potential of the caspase inhibition. Potentially, the strong increase in IFN secretion upon triple-treatment with irradiation, ATR inhibition and caspase inhibition may outweigh the concomitant loss of HMGB1 release, thus resulting in an overall increased antitumor immune response. Notably our finding of caspase-dependent HMGB1 release is consistent with results in *e.g.* apoptosis-mediated sepsis (42) and for macrophages treated with a proteasome inhibitor (43). Furthermore, our results suggest that caspase inhibition does not always abolish the HMGB1 release, as shown for H1975 cells where the caspase inhibition also caused phosphorylation of MLKL, a necroptosis marker. As mentioned above, the results in H1975 resemble the previous report of necroptosis and HMGB1 release after caspase inhibition and irradiation in mouse melanoma cells (32). Triple-treatment with caspase inhibition, irradiation and ATR inhibition can thus likely induce necroptosis-dependent HMGB1 release in some cases. Interestingly, our finding that ATP secretion is increased by caspase inhibition is in contrast to previous studies showing caspase-dependent ATP secretion during chemotherapy-induced ICD (28). The co-treatment with irradiation and ATR inhibition thus likely activates an alternative, non-apoptotic mechanism of ATP secretion. Indeed, ATP secretion independent of the apoptosis mediators BAX and BAK has been reported in cells with intact plasma membrane (16). In line with this, the measured ATP in our experiments most likely reflects secretion from live cells, as it was high already at 48 hours after treatment and was increased when the viability was increased by caspase inhibition.

To accurately measure the surface-presentation of CALR, we included a unique barcoding strategy in our live-cell flow cytometry assay. Previous studies that have used flow cytometry to measure ecto-CALR have also included a dye to distinguish live from dead cells (*e.g.* (44, 45)), similar to the use of propidium iodide in our assay. However, we are not aware of any previous study that has included a similar barcoding strategy for ecto-CALR measurements. By including barcoding with the membrane-permeable Hoechst 33342 dye, the CALR signal of each sample can be normalized to the CALR signal of a common live-cell standard. As the Hoechst-stained cells are added to the samples prior to antibody staining, this procedure eliminates any potential variation due to *e.g.* differences in antibody concentration or cell numbers between the samples. Notably, the background signals of the secondary antibody controls increased upon treatment with irradiation and/or ATR inhibition. As a similar increase in background signals was seen for non-stained cells (data not shown), this most likely reflects increased autofluorescence due to treatment-induced changes to the cell. This increase in background signals particularly becomes important when measuring the expression of low-abundance surface proteins, such as ecto-CALR. When measuring ecto-CALR it is thus necessary to accurately obtain the background signal for each treatment. In our optimized flow cytometry protocol, we measure the background signals in aliquots taken from every sample, which then also contains the Hoechst-stained mock cells. We thus obtain a highly accurate measurement of the background signals.

Previous studies have shown that radiation treatment alone can induce ICD, as measured by several DAMPs (12, 46). This is further substantiated in our study. We detected radiation-induced increases in both HMGB1 release, ATP secretion and ecto-CALR in several cell lines. Notably the responses appear to vary between cell lines, as HMGB1 release and ATP secretion were not detected after irradiation alone in U2OS cells. Interestingly, this difference between U2OS and the other cell lines was not likely caused by a corresponding difference in radioresistance. The amount of non-viable cells after irradiation was not markedly lower for U2OS than the other cell lines (**Supplementary Figure S1**), and previous studies have shown largely similar clonogenic survival for U2OS, H460 and H1975 after irradiation (47, 48). Treatment with ATR inhibitor alone also gave detectable increases in HMGB1 release and ATP secretion, but only for the highest concentration of VE822 (250 nM) at 72 hours post treatment, and not for AZD6738. The effects of the co-treatment could thus not be explained by the effects of ATR inhibition alone. Importantly, the combined treatment with irradiation and ATR inhibition caused increased HMGB1 release and ATP secretion compared to mock in all cell lines tested. The ultimate functional endpoint of ICD is the priming of tumor-specific T cell responses, mediated through recruitment of antigen-presenting cells to the tumor microenvironment. Although it has been reported that simultaneous presence of every ICD hallmark factor is crucial for ICD *per se* (49), it is reasonable to assume that it is the total immunogenicity of the microenvironment – contributed by the concoction of many different DAMPs – that governs the functional endpoint. Hence, lack of response for some of the hallmarks, such as ecto-CALR in this study, does not rule out the immunogenic potential, as long as there is adequate presence of other immunogenic factors.

The immunostimulatory effects of ATR inhibition in combination with irradiation may potentially be exploited to improve the efficacy of immune checkpoint blockade. Indeed, triple-treatment with radiotherapy, ATR inhibitor and anti-PD-L1 antibodies have been shown to increase antitumor immunity in preclinical mouse models. In a hepatocellular carcinoma model, the triple-treatment caused increased CD8^+^ T cell infiltration, less regulatory T cells and increased immunological memory compared to after co-treatment with radiotherapy and anti-PD-L1 (21). Similarly, CD8^+^ T cell infiltration was increased after triple-treatment of murine colorectal cancer models (50). Another study found that the activity of natural killer (NK) cells was boosted by immune checkpoint blockade (targeting either PD-1 or T cell immunoreceptor with Ig and ITIM domains (TIGIT)) in combination with ATR inhibition and radiotherapy in a murine oral squamous cell carcinoma model (51). Moreover, analogous to the combination studies with radiotherapy, long-lasting antitumor immunity was also observed in a murine colorectal model when ATR inhibition was combined with anti-PD-L1 antibodies and platinum-based chemotherapy (52). Promising preclinical results have led to several ongoing early-phase clinical trials with immune checkpoint blockade in combination with ATR inhibitors, and at least one of these studies addresses the triple-treatment with radiotherapy [reviewed in (11, 53)]. Of note is that even the co-treatment of radiotherapy and immune checkpoint blockade is far from fully developed. Both the optimal radiation dose and timing and sequence of treatment remain to be determined [reviewed in (54–56)]. The optimization of the triple-treatment is even more complex. Interestingly, it was recently shown that prolonged ATR inhibitor treatment can abolish the antitumor immune responses in two murine cancer models (colorectal CT26 and melanoma B16-F10). A short-term ATR inhibitor treatment and subsequent cessation was required to increase CD8^+^ T cell responses to radiotherapy and immune checkpoint inhibitors (57).

In conclusion, our results substantiate the potential for ICD induction by radiotherapy, and show that irradiation in combination with ATR inhibition further increases this potential. Induction of ICD may thus likely contribute, at least to some extent, to the immunostimulatory properties of such combined treatment. Moreover, our results show distinct roles of caspase activation in the regulation of each ICD hallmark. Further studies revealing the exact immunomodulating mechanisms induced by irradiation and ATR inhibition may help to develop new biomarkers for treatment response and to optimize treatment schedules. Understanding these mechanisms will also likely help to further exploit the immunostimulatory properties of the combined treatment, *e.g. via* subsequent treatment with immune checkpoint blockade.

## 4 Materials and methods

### 4.1 Cell culturing and treatment

Human U2OS osteosarcoma and H460 NSCLC cells were grown in Dulbecco’s modified Eagle’s medium with GlutaMAX-I (Gibco by Life Technologies, ThermoFisher Scientific #61965059), and human H1975 and SW900 NSCLC cells were grown in Roswell Park Memorial Institute 1640 medium with GlutaMAX-I (Gibco by Life Technologies, ThermoFisher Scientific #61870044) in a humidified 5% CO_2_ atmosphere at 37°C. Both media were supplemented with 10% foetal bovine serum (FBS, Biowest #S1810) and 1% penicillin–streptomycin solution (10,000 IU/ml; 10,000 µg/ml) (Pen Strep, Gibco by Life Technologies, ThermoFisher Scientific #15140122). Cell line identity was confirmed by short tandem repeat analysis, and the cultures were tested for *Mycoplasma* infection. The cells were treated with ATR inhibitors VE822 (berzosertib/VX970, Selleckchem #S7102) at 250 nM or 50 nM and AZD6738 (ceralasertib, Selleckchem #S7693) at 1250 nM, and the pan-caspase inhibitor Q-VD-OPh (quinoline-Val-Asp-difluorophenoxymethylketone, MedChemExpress #HY12305) at 10 µM, for 10-30 minutes before X-irradiation (160 kV Faxitron Corporation CP-160, dose rate 1 Gy/min). Caspase-1 inhibitors Ac-YVAD-cmk (acetyl-Tyr-Val-Ala-Asp-chloromethylketone) and VX-765 (belnacasan) (both from InvivoGen, #inh-yvad and #inh-vx765i-1, respectively) were employed at 60 and 120 µM.

### 4.2 Western blotting of released HMGB1 in growth medium supernatants

For measuring extracellular HMGB1, an equal number of cells were seeded in 6 cm dishes for all samples within an experiment. Cells were treated as indicated and incubated for 72 hours, before the growth medium supernatants were harvested. The medium was centrifuged at 12100 × *g* for 5 minutes for exclusion of cells and debris, and the resulting supernatants were diluted 1:2 in 5X loading buffer (Pierce Lane Marker Reducing Sample Buffer, ThermoFisher Scientific #39000) and boiled at 95°C for 10 minutes. The samples were loaded onto SDS polyacrylamide 4-15% gradient gels (Mini-Protean TGX, Bio-Rad #4561086) for electrophoresis, and blotted onto nitrocellulose membrane (Bio-Rad #1704270). The membrane was stained with Ponceau S (Sigma-Aldrich #P7170), and blocked in 5% non-fat skimmed milk (Sigma-Aldrich #70166) in phosphate-buffered saline with 0.1% Tween-20 (Bio-Rad #1610781) (PBST). The membrane was stained with anti-HMGB1 antibodies (Abcam, ab18256, 1:2000 in blocking solution) at 4°C over-night, and thereafter stained with horseradish-conjugated secondary antibodies (Jackson ImmunoResearch, #111-035-144, 1:10 000 in blocking solution) for minimum 30 minutes before addition of enhanced chemiluminescence (ECL) solution (SuperSignal West, ThermoFisher Scientific #34580/#34076/#34095) and processing (ChemiDoc MP, Bio-Rad). Quantification was performed in Image Lab 4.1 (Bio-Rad). For blotting of caspases in the corresponding cell lysates, cells were washed with PBS and stored at -80°C. The cells were lysed with whole-cell lysis buffer [20 mM NaCl, 2 mM MgCl_2_, 50 mM Tris-HCl pH 7.5, 0.5% Triton X-100 (Sigma-Aldrich #T9284)] with protease and phosphatase inhibitor cocktails (cOmplete mini (EDTA-free) and PhosSTOP EASYpack, Roche, Sigma-Aldrich #5892791001 and #4906837001) and benzonase (100 IU/ml; Merck/Sigma-Aldrich #70664-3). The lysates were diluted based on protein concentration measurements (Micro BCA Protein Assay kit, ThermoFisher Scientific #23235), before 5X loading buffer was diluted 1:4 in each sample. The samples were boiled before SDS-PAGE (Criterion TGX Stain-Free gels, Bio-Rad #5678085) and immunoblotting as described above. *Primary antibodies*: Cleaved Caspase-3 (Asp175) (5A1E), 1:100, Cell Signaling Technology #9664. PARP1 (F2), 1:200, Santa Cruz Biotechnology #sc-8007. MLKL phospho-Ser358 (D6H3V), 1:1000, Cell Signaling Technology #91689. MLKL (D2I6N), 1:1000, Cell Signaling Technology #14993. γ-tubulin, 1:1000, Sigma-Aldrich #T6557. Quantification of HMGB1 blots were performed by use of loaded volume gradients (see *e.g.* 50%, 25%, 10% in **Figure 4A**).

### 4.3 Immunofluorescence microscopy analysis of HMGB1 release

Cells were seeded (3·10^5^ cells for treatments, 1·10^5^ for mock) in 6 cm dishes containing glass coverslips, and incubated over-night. The samples were treated as indicated, and incubated for 72 hours. The coverslips were washed with phosphate-buffered saline (PBS) and the cells were fixated with a 10% formalin solution (Sigma-Aldrich #HT5011) for 10 minutes. The cells were washed three times in PBS, and stained with Alexa Fluor 594-conjugated wheat germ agglutinin (WGA) (1:1000, ThermoFisher Scientific #W11261) for 10 minutes. The cells were washed three times in PBS and permeabilized with 0.2% Triton X-100 (Sigma-Aldrich #T9284) in PBS for 5 minutes. The cells were washed and stained with anti-HMGB1 antibodies (Abcam, ab18256, 1:1000 in growth medium with 10% FBS) for 1 hour, followed by three washes in PBS and secondary antibody staining (Molecular Probes by Life Technologies (ThermoFisher Scientific #A-21206), Alexa Fluor 488, 1:1000 in growth medium with 10% FBS) for 30 minutes. The cells were washed, stained with 0.6 µg/ml permeable Hoechst 33342 (Invitrogen, ThermoFisher Scientific #H3570) in PBS for 5 minutes and eventually mounted onto object slides with mowiol solution (Mowiol 4-88, Sigma-Aldrich #81381).

### 4.4 Flow cytometric analysis of viability and intracellular HMGB1

Cells were harvested by trypsinization and centrifuged at approx. 400 × *g*. Resulting cell pellets were stained with Pacific Blue (0.0375 ng/µl final concentration, *V* = 200 µl), and incubated at 4°C for 15 minutes. The samples were washed with 3 ml PBS/1% FBS, and centrifuged as before. For the viability measurements presented in **Supplementary Figure S1**, the resulting cell pellets were thereafter fixated with 70% EtOH, and stored at -20°C. For intracellular staining of HMGB1, the cell pellets were fixated in 10% formalin solution (Sigma-Aldrich #HT5011) for 10 minutes at room temperature, before they were washed in PBS, resuspended in 70% EtOH and stored at -20°C. An aliquot of a barcode-stained (succinimidyl ester-conjugated Alexa Fluor 647, ThermoFisher Scientific #A20006) mock sample was added to all samples, similarly as before (*e.g.* (47)), allowing accurate quantification of HMGB1 levels. The samples were washed with PBS/2% FBS, and the cell pellets were stained with primary anti-HMGB1 antibodies (Abcam, ab18256, 1:500 in flow cytometry staining buffer [0.1% IGEPAL CA-630 (Sigma-Aldrich #I3021), 6.5 mM Na_2_HPO_4_, 1.5 mM KH_2_PO_4_, 2.7 mM KCl, 137 mM NaCl, 0.5 mM EDTA pH 7.5)] for 1 hour and secondary anti-rabbit Alexa Fluor 488 (Molecular Probes by Life Technologies (ThermoFisher Scientific #A-21206), 1:500 in flow cytometry staining buffer) for 30 minutes. The samples were thereafter analysed by flow cytometry (BD LSR II, BD Biosciences). Subsequent analyses were conducted with FlowJo v10.

### 4.5 *CellTiter-Glo* detection of secreted ATP in growth medium supernatants

Cells were treated with radiation and ATR inhibitors as described and incubated until 6 hours before harvest. The growth media were aspirated, before the dishes were washed with PBS, and then given 1 ml serum-free medium (with inhibitors at given dose, if used). The samples were incubated for the remaining 6 hours – of which ATP secretion would be detected – before the growth media were harvested. The medium supernatants were centrifuged at 12100 × *g* for 5 minutes, and the resulting supernatants were transferred to new tubes. The supernatants were centrifuged a second time at 12100 × *g* – to ensure exclusion of any floating cells – and the resulting supernatants were loaded onto a 96-well plate with clear bottoms and white walls (Corning Costar 3610, Sigma-Aldrich #CLS3610-48EA), together with samples for an ATP standard curve. The samples subsequently underwent the *CellTiter-Glo* procedure after the supplier’s protocol (CellTiter-Glo Luminescent Cell Viability Assay, Promega #G7572), before spectrophotometric analysis.

### 4.6 Live-cell flow cytometric detection of surface-presented calreticulin

Cells were seeded and let adhere over-night, before the cells were treated as described, and incubated for 24 or 72 hours. The dishes were harvested – both growth medium supernatants and adhered cells – by use of TrypLE Express (Gibco by Life Technologies, ThermoFisher Scientific #12563029). First, the mock sample was centrifuged at approx. 400 × *g* (2000 rpm). The cell pellet was resuspended in 100 µl medium with 1 µg/ml Hoechst 33342 (Invitrogen, ThermoFisher Scientific #H3570) and incubated at room temperature for 30 minutes, for barcode staining. The barcode-stained sample was thereafter washed with PBS/1% FBS and resuspended in PBS/1% FBS. Meanwhile, the remaining samples were harvested. Equal aliquots of the barcode-stained mock samples were thereafter added to each of the remaining samples, before these were split in two for subsequent primary antibody staining and secondary antibody control staining. The samples were centrifuged at approx. 500 × *g*, and the cell pellets were resuspended in 100 µl medium (10% FBS) with primary anti-CALR antibodies (Abcam, ab2907, 1:250), or plain medium (10% FBS) for secondary antibody controls, and incubated on ice for 30 minutes. The samples were washed, and resulting cell pellets were resuspended in 100 µl medium (10% FBS) with secondary antibodies (Molecular Probes by Life Technologies (ThermoFisher Scientific #A-21206), Alexa Fluor 488, 1:500). The samples were incubated on ice for 30 minutes, and washed. The samples were resuspended in PBS and transferred to flow cytometry tubes. 1 µl propidium iodine (1.667 mg/ml) was added to the samples 2 minutes prior to flow cytometry (BD LSR II, BD Biosciences), for live/dead staining. Flow cytometric analysis was conducted in FlowJo v10. Ecto-CALR signals were calculated by [(signal_treated_ – background_treated_)/(signal_mock_ – background_mock_)], where the secondary antibody control signals constitute the background values. Median Alexa Fluor 488 values were used as outputs from the flow cytometry.

### 4.7 Statistics

For measurements with ≥ 3 replicates, results are presented with standard error of the mean (SEM) error bars. Dots in bar charts indicate individual experiments. *p* values (two-tailed, one-sample Student’s *t* test for pairs involving normalization value; two-tailed, paired-samples Student’s *t* test for the remaining pairs) were calculated with IBM SPSS Statistics v28, with significance level set to 0.05. *p ≤ 0.05, **p ≤ 0.01, ***p ≤ 0.001.

## Data availability statement

The raw data supporting the conclusions of this article will be made available upon request to the corresponding author.

## Author contributions

Conceptualization: RS, AEM, SH. Experiments: AEM, SH, KK. Data analysis: AEM, SH, KK, RS. Supervision: RS, SH. Critical review of work: All authors. Writing – original draft preparation: AEM, RS. Writing – editing: All authors. Funding acquisition: RS. All authors contributed to the article and approved the submitted version.
