# Supplementary material for: Immunogenic cell death after combined treatment with radiation and ATR inhibitors is dually regulated by apoptotic caspases
Source: Front Immunol. 2023 Jun 6;14:1138920. doi: 10.3389/fimmu.2023.1138920 (PMC10279842; doi:10.3389/fimmu.2023.1138920)
Supplement: Supplementary file 1 [file DataSheet_1.pdf]

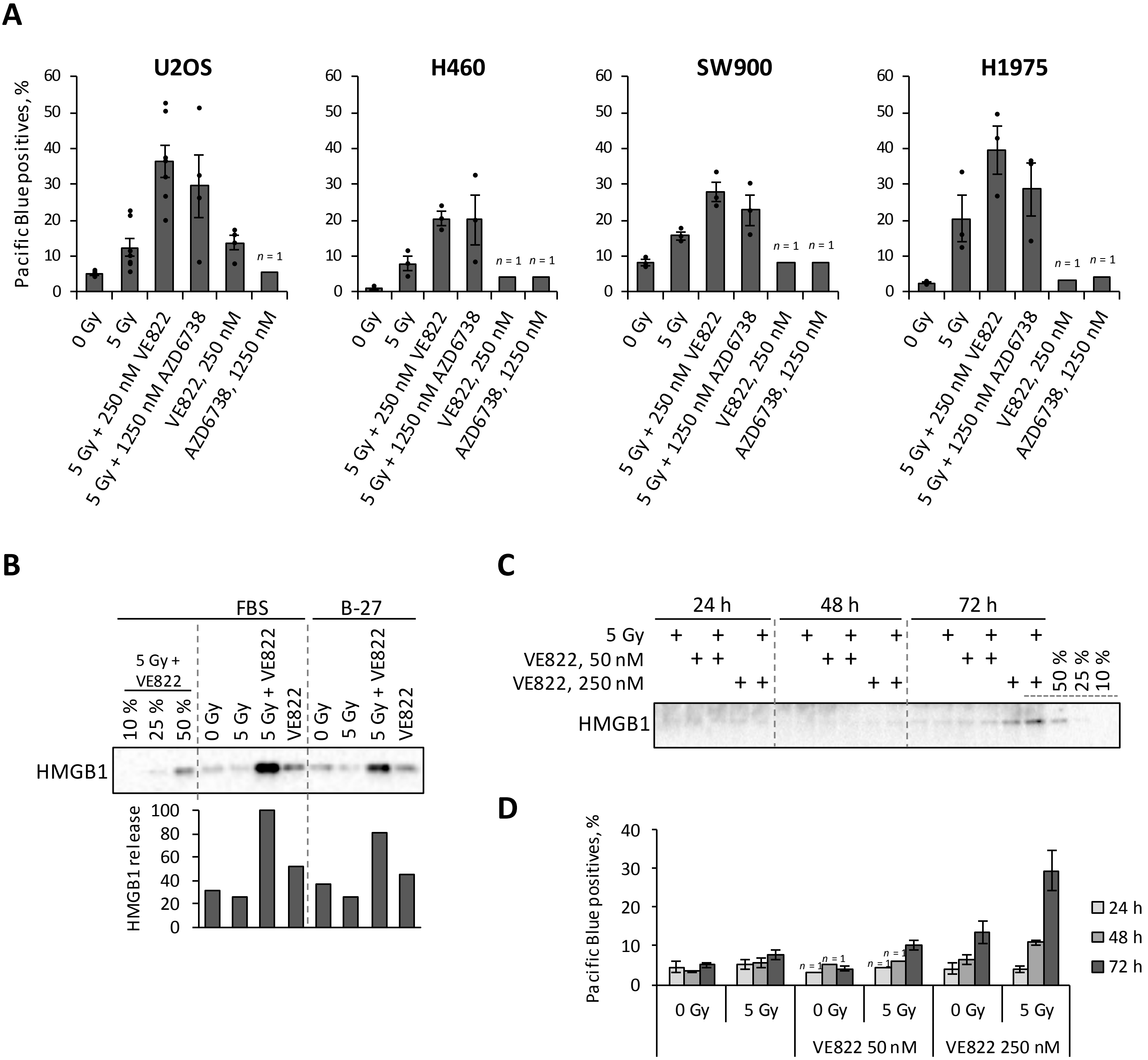

Supplementary figure S1. A) Percentage of non-viable cells (as defined by uptake of Pacific Blue stain) at 72 hours post indicated treatments in U2OS, H460, SW900 and H1975 cells, measured by flow cytometry. **B)** HMGB1 release (measured as in Figure 1) from U2OS cells cultured in medium with FBS and in serum-free medium with the serum substitute B-27. VE822 was employed at 250 nM. Quantification of relative HMGB1 levels in the media (bar chart) was conducted with use of the gradient shown to the left in the immunoblot. Findings were verified by another independent experiment. **C)** Immunoblot showing the HMGB1 released from U2OS cells to the growth medium at different time-points after indicated treatments. **D)** The percentage of non-viable cells in experiments as in (C), measured by flow cytometry as in (A). (Data-points for the 72 hours time-point are also included in the U2OS plot in (A)).

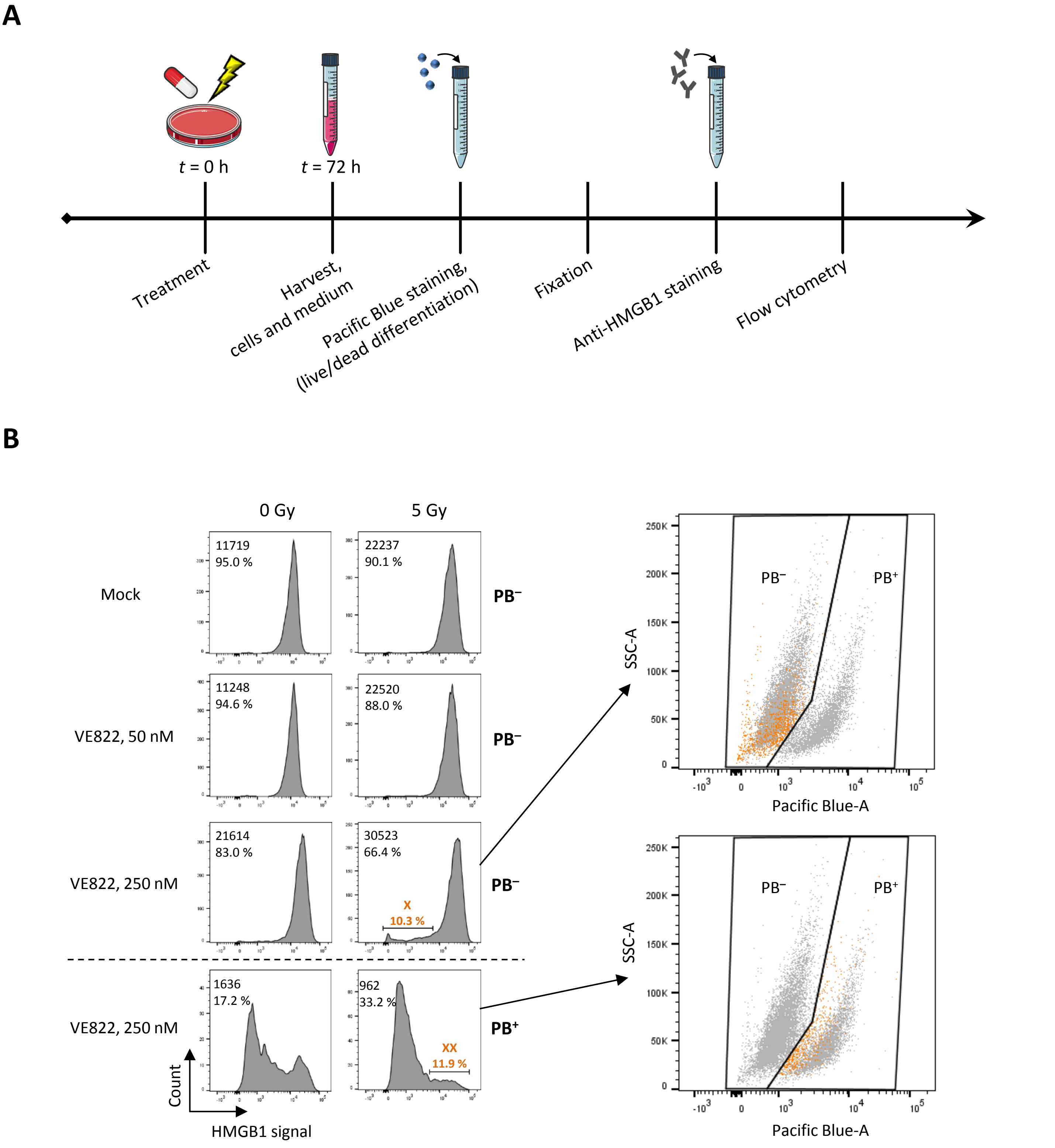

Supplementary figure S2. A) Experimental set-up for assessment of HMGB1 levels in viable *versus* non-viable cells. Cell samples were harvested at 72 hours post treatment and stained with Pacific Blue (PB), to indicate lack of plasma membrane integrity (non-viability), before the cells were fixated with formalin, stained with anti-HMGB1 and secondary antibodies and analyzed by flow cytometry. **B)** Histograms of intracellular HMGB1 levels in viable (PB^-) and non-viable (PB^+) U2OS cells. Note that for the sample treated with 5 Gy + 250 nM VE822, viable cells (PB^-) have a high-signal peak with a low-signal tail, whereas non-viable cells (PB^+) have a low-signal peak with a high-signal tail. This suggests that HMGB1 release is a process that mainly occurs in non-viable cells (probably due to rupture of the plasma membrane), but also to some degree occur in viable (but dying) cells prior to loss of plasma membrane integrity. Numbers in histograms represent median HMGB1 value and population size (in percentage of total number of single cells). Cells constituting the ‘tail values’ (labelled X and XX in the histograms) are indicated with orange color in the dot plots to the right. Results are shown for a representative experiment (n = 3).

A

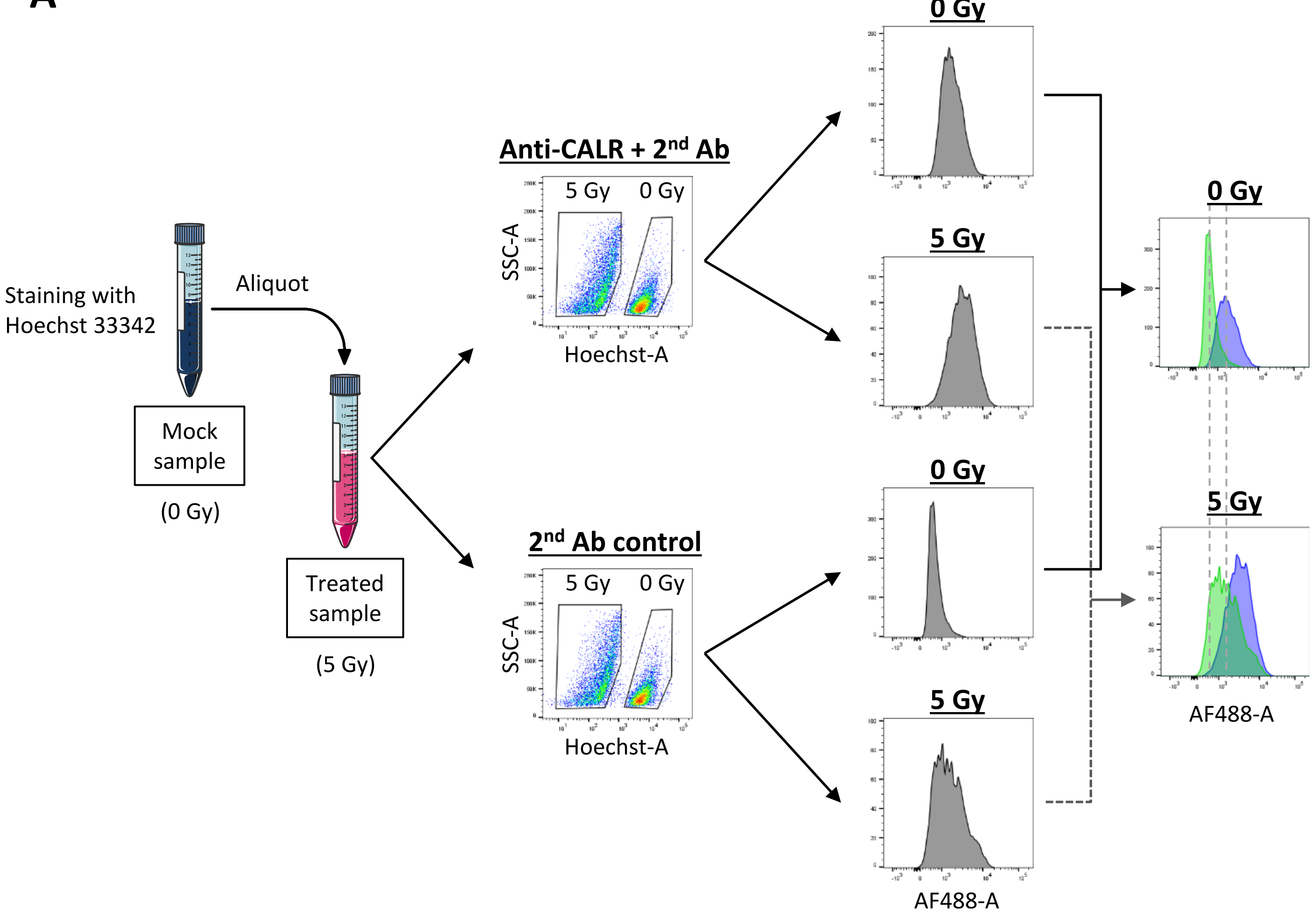

B

	Anti-CALR + 2nd Ab	2nd Ab control
	**Median, AF488**	**Median, AF488**
**0 Gy**	1636	628
**5 Gy**	2957	1392

C

$$\text{Signal}_{\text{ecto-CALR}} = \frac{\text{Signal}_{5\text{ Gy}} - \text{Background}_{5\text{ Gy}}}{\text{Signal}_{0\text{ Gy}} - \text{Background}_{0\text{ Gy}}} = \frac{2957 - 1392}{1636 - 628} = \mathbf{1.55}$$

Supplementary figure S3. ***Exemplified calculation of normalized ecto-CALR signals*****. A)** Flow chart demonstrating how Alexa Fluor 488 (AF488) median values were extracted from each of the barcoded populations of both the primary antibody-stained fraction and the secondary antibody (2nd Ab) control fraction from each of the treated samples. The rightmost histograms demonstrate shifts in background signals (green) and primary antibody signals (blue) between the mock population and the treated population. **B)** AF488 median values from each of the histograms shown in (A), for further use in calculation of ecto-CALR signal. **C)** Example of calculation of ecto-CALR signal normalized to the internal mock control, demonstrated with values from (A) and (B). Data material used in (A-C) is from one of the data point for H1975 presented in Figure 3C.

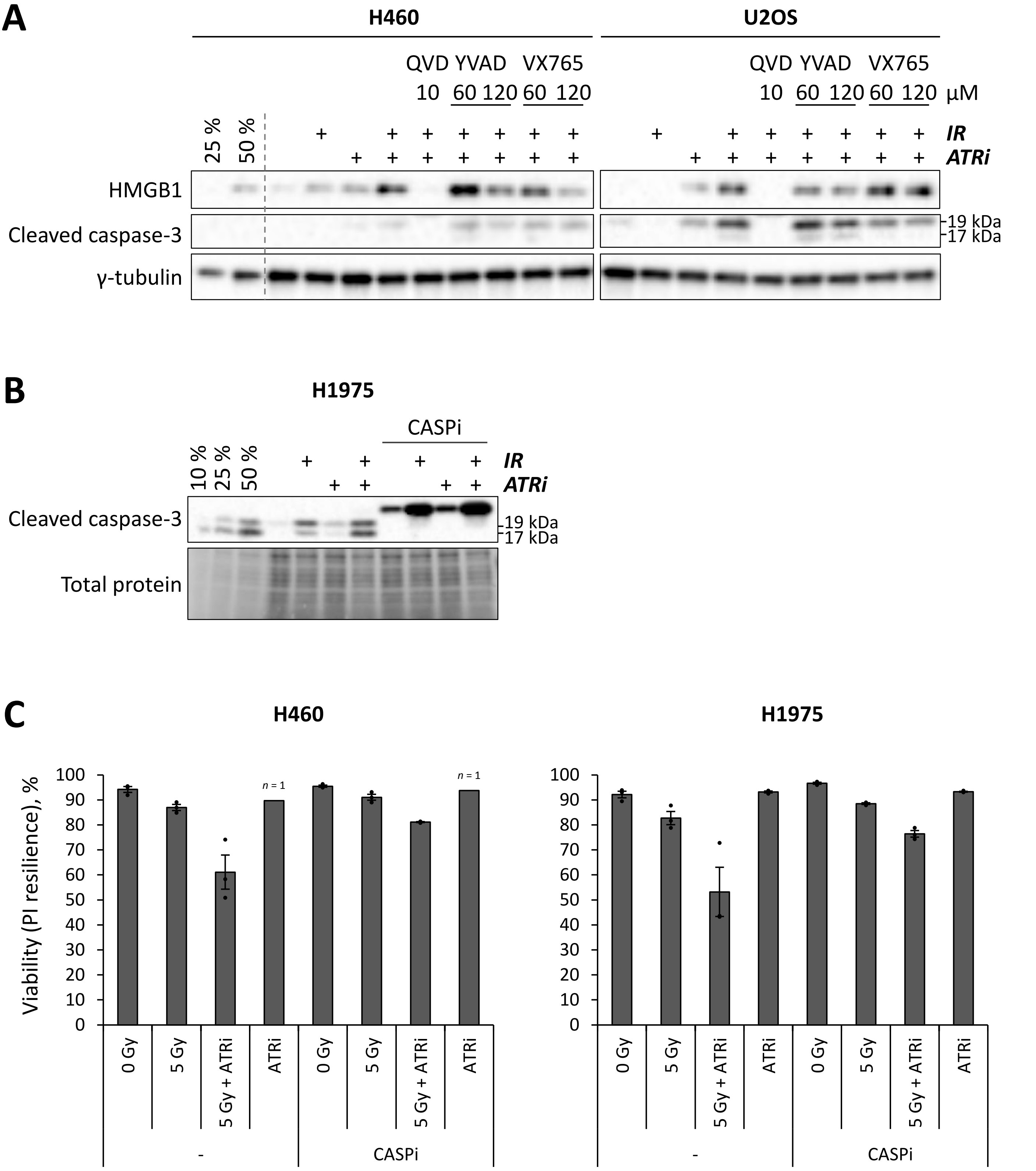

Supplementary figure S4. A) Immunoblots of extracellular HMGB1 in medium supernatants (top), and of cleaved caspase-3 and γ-tubulin in corresponding lysates (bottom), from H460 and U2OS cells 72 hours after treatment with 5 Gy radiation (IR), 250nM VE822 (ATRi) and the pan-caspase inhibitor Q-VD-OPh (QVD), the caspase-1 inhibitor Ac-YVAD-cmk (YVAD) or the caspase 1 and 4 inhibitor VX-765 (VX765), as indicated. The gradient to the left is from the co-treated sample. Similar results were obtained in >2 additional experiments. **B)** Immunoblot analysis in H1975 from an independent experiment performed as in Figure 4C, to verify lack of caspase-3 cleavage after addition of the pan-caspase inhibitor Q-VD-OPh (10 µM; CASPi). Gradient to the left is from the co-treated sample. **C)** The percentage of viable cells based on propidium iodide exclusion (PI resilience) for H460 and H1975 cells treated as indicated, with and without pan-caspase inhibitor Q-VD-OPh (CASPi, 10 µM). Results are extracted from the ecto-CALR assays conducted for Figure 4E.
